# Effects of the Counseling Program on Strengthening Mental-Crisis Management to Strengthen Adolescent Life Crisis Awareness

**DOI:** 10.2174/17450179-v18-e2208110

**Published:** 2022-09-20

**Authors:** Angkana Jirarode, Pisit Rungrojwatanasiri

**Affiliations:** 1 Department of Mental Health and Psychiatric Nursing, Faculty of Nursing, Thammasat University, Pathumthani, Thailand

**Keywords:** Counseling program, Strengthening, Mental-crisis management, Capacity, Health-crisis awareness, Adolescents

## Abstract

**Background:**

A mental crisis is a dangerous state with many subsequent harmful effects on the adolescent. It is a state in which the adolescent needs help and must get that help immediately. If they do not receive the assistance they require, they may end up isolating themselves from social interaction and become completely preoccupied with themselves. The counseling program is therefore being put into effect to strengthen the mental crisis management capacity of adolescents and to offer counseling and assistance to students who find themselves beset with a mental crisis so that they will be fully able to manage their mental crises, restore a sense of balance to their psyches, and carry on with their lives effectively.

**Objective:**

The aim of the study was to compare the life crisis awareness scores of adolescent students enrolled in a program of study at Thammasat University.

**Methods:**

This research was quasi-experimental and consisted of a two-group pretest-post test design. They were subdivided into two groups of 30 students each by simple random sampling, of which one group was the experimental group and the other was control group. The experimental group underwent a counseling program of bolstering mental crisis management capacity once a week for a period of six weeks. Results were assessed using a crisis awareness evaluation form. Results were assessed twice, before testing and after testing. The data were analyzed by the statistics of percentage, mean, dependent t-test, and independent t-test.

**Results:**

1. The average life crisis awareness scores of the adolescents in the experimental group following acceptance into the counseling program by fortifying their mental crisis management capacity (M=132.77, SD=11.03) were higher than they were prior to program entry (M=113.73, SD=14.40), with a statistical significance (t=6.81, p<0.001).

2. The average life crisis awareness scores of the experimental group that had been accepted into the above-mentioned counseling program were higher after undergoing testing (M=132.77, SD=11.03) than the scores for the control group (M=118.83, SD=10.73), with a statistical significance (t=4.95, p<0.001).

**Conclusion:**

This program thus helps the adolescent restore a sense of mental balance and live a normal happy life.

## 1 INTRODUCTION

Adolescence is a stage at which the developing young adult is forced to deal with changes in a number of areas, including the body, mind, emotions, and society [1, 2]. Whenever an adolescent must confront a life crisis and is unable to manage it well, that young person goes into a state of prolonged misery that severely upsets his (or her) daily life functions, particularly in the areas of school and work, and relationships with other people. The life crisis is a state that the individual must combat to secure his (or her) mental balance and make adjustments in himself (or in herself) in the days ahead [3, 4]. An adolescent life crisis usually develops from four principle causes, namely: 1) pressure from oneself, 2) pressure from the family, 3) pressure from school, and 4) pressure from society. The majority of crises will generally develop instantly and tend to last for about four to six weeks. Depending on the event being confronted, a crisis can generate overwhelming feelings of emotion and an elevation of stress, as well as feelings of hopelessness with no sense of direction. The individual in this situation becomes utterly confused [5-7].

The impact of a life crisis not only affects one’s own self, that person’s family and his (or her), work but also causes the individual thus affected to lose any sense of self-worth that he (or she) may have had. Consequently, the adolescent lapses into a mental crisis. When the adolescent is unable to confront his (or her) problems and make the necessary adjustments in himself (or in herself) to get through the crisis, that person becomes very dissatisfied with himself (or with herself) and generates a feeling of being no good. That person then becomes worn out and no longer functional, and lapses into a state of despair, unable to handle regular daily tasks [6-8]. He (or she) may go so far as to inflict violence on himself and his family (or on herself and her family), both physically and verbally, such as by hitting, accusing, cursing or deliberately ignoring them. The impact on that person’s schooling is a decrease in learning ability and a subsequent drop in learning effectiveness from sheer boredom and combined physical and mental exhaustion [3, 9].

A mental crisis is a dangerous state with many subsequent harmful effects on the adolescent. It is a state in which the adolescent needs help and must get that help immediately. Although a mental crisis is an experience that develops normally in a person’s life and may not be indicative of an illness or show any clear symptoms, it is still considered a crisis of emotions that has developed, which the adolescent must resolve to overcome to maintain a sense of balance between himself and his surroundings [10, 11]. If, however, the adolescent is unable to confront and manage these life crisis problems, the result will be a problematic chain reaction that will impact his family and other involved individuals. It will cause an absence of any happiness and an increase in stress levels. Furthermore, the affected adolescent is apt to become emotional and violent in dealing with his problems, thus aggravating his mental crisis even further [8, 9].

There is a direct impact upon the adolescent who must confront a mental crisis that has suddenly developed but is unable to manage. If they do not get the kind of help their need, they may, as a result, become totally lost in thought over themselves and isolate themselves from any interaction with others [3, 12]. They will feel that they are unproductive and worthless and will develop an inferiority complex, leading eventually to mental health problems or mental illness. The psychological reaction of someone undergoing a mental crisis will be different in each case [2, 3]. Even those who have endured the same event may have mental crises that are unequal in their level of severity. Those who have been heavily impacted will be spending a lot of time in their development and treatment for their emotional reactions that include worrying, fear, depression, anger, irascibility, desire for revenge, self-blame, blaming of others, hopelessness, and emotional instability [12, 13]. These symptoms may develop after having experienced a crisis. The crisis may emerge and then decrease within a period of no more than two months. If these symptoms persist, it is essential that the patient see a person with the right expertise for additional help [14].

The mental remedy for someone who has experienced a mental crisis consists in providing targeted psychological care for the care support of that person and the prevention of any disease or psychiatric illness that would follow in the absence of care [8, 12]. The theory of cognitive behavior therapy (CBT) of Beck *et al*. (1979) is focused on solving problems that are current and offsetting twisted notions with the purpose of lowering the level of physical and mental misery [15]. The concepts of Conner and Butterfield (2003) are based on their belief that by relieving their mental suffering, adolescents will help reduce their feelings of pain or guilt that are disturbing them and lower their stress levels [16]. They will thereby have better thoughts and feelings, increase their capacity or ability in their own problem management, and make the necessary adjustments in themselves so they can return to life or normality and happiness [15, 16]. Adolescents will also increase their mental crisis management capacity whenever they are challenged by another life crisis that may emerge in the future [15, 17]. Providing assistance and counsel that would be appropriate to an adolescent who is confronting a mental crisis will help that person to ease his or suffering and thus reduce any annoying feelings of illness or guilt. His thoughts and feelings will then be so much better [18-20].

Researchers in their capacity as instructors and experts in the area of mental health and psychiatric care are in a position to provide counseling assistance to an adolescent undergoing a mental crisis. In particular, bachelor-level students from the Department of Nursing Science and various other departments at Thammasat University found that students with mental health problems who are dealing with a mental crisis receive counseling upon request. The majority of them develop a mental crisis from disappointment in their studies, their love life, their relationships, and self-adjustment [21]. Researchers are thus focusing their attention on the psychological care of the adolescent student group at Thammasat University. Their purpose is to revive the mental functioning of this student group so that they may be able to manage their own mental crises all on their own, restore a sense of balance to their psyches, and carry on with their lives effectively. This counseling program is therefore being put into effect to strengthen the mental crisis management capacity of adolescents and to offer counseling and assistance to students who find themselves be set with a mental crisis [22, 23]. It is meant to revive the mentality of this adolescent student group so that they will be fully able to manage their own mental crises, restore a sense of balance to their psyches, and carry on with their lives effectively [23].

### 1.1 Overall Research Objectives

This research is mainly intended to study the results of the counseling program for strengthening the mental crisis management capacity of adolescent students at the Thammasat University (Rangsit Campus).

#### 1.1.2 Specific Objectives

(1) To compare the life crisis awareness of adolescents in the experimental group both before and after acceptance into the counseling program for the strengthening of their mental crisis management capacity.

(2) To compare the life crisis awareness of adolescents in the experimental group after entry into the counseling program for the strengthening of their mental crisis management capacity with the mental crisis awareness of the control group adolescents, who received regular conventional care.

### 1.2 Research Questions

(1) Is the average life crisis awareness score of the adolescents in the experimental group, following their entry into the counseling program for the strengthening of their mental crisis management capacity, higher than it was before they underwent the program testing procedure?

(2) Is the average life crisis awareness score of the adolescents in the experimental group, following their entry into the counseling program for the strengthening of their mental crisis management capacity, higher than the average score of the control group?

### 1.3 Hypotheses

(1) The average life crisis awareness score of the adolescents in the experimental group, following their entry into the counseling program for the strengthening of their mental crisis management capacity, is higher than it was before they underwent the program testing procedure.

(2) The average life crisis awareness score of the adolescents in the experimental group, following their entry into the counseling program for the strengthening of their mental crisis management capacity, is higher than the average score of the control group, whose members received regular conventional care.

### 1.4 Conceptual Framework of Research

The present research applied the theory of CBT of Beck *et al*. (1979), which focuses on present-day problem solving and the resetting of distorted thought patterns [15]. Its purpose is to develop counseling guidelines for the strengthening of the mental crisis management capacity of adolescent students who are experiencing a mental crisis to reduce their physical and psychological misery. It combines the concepts of Conner and Butterfield (2003), who believed that relieving an adolescent of his misery helps to reduce his feelings of pain or guilt that may be bothering him, allowing him to think better and to feel better, to lower his stress level, and to increase his capacity or ability to manage his own problems [16]. The adolescent is thus able to adapt to a normal, happy life. It also helps to increase the mental crisis management capacity of adolescent students when they are forced to confront a life crisis that may resurface in Fig. (**1**).

## 2 MATERIALS AND METHODS

The format of this research was quasi-experimental and consisted of a two-group pretest-posttest design. Its objective was to compare the life crisis awareness scores of adolescents after acceptance into a counseling program for strengthening the mental crisis management capacity of adolescent students enrolled in a program of study at the Thammasat University (Rangsit Campus). It was carried out in groups of six weeks each once a week for 60-90 minutes over a six-week period in conjunction with the standard conventional care of the University. Results were measured both before and after the testing phase by research assistants.

### 2.1 Population and Sample

(1) Sample groups included adolescent students who were studying at Thammasat University. Sample-group size was calculated by use of the software package G*power, version 3.1.9.2. The effect size of .80, the confidence level was 85%, and the tolerance was 0.05, A sample total of 48 people was obtained. The sample group was then increased in size by 25% to guard against possible drop-outs during the research, arriving at a sample size of 60 people by the use of a simple random sampling technique. The sixty participating individuals were then partitioned into an experimental group of 30 people and a control group of 30 people. Simple random sampling was used by randomly selecting students who came for counseling services on Monday, Wednesday, and Friday.

(2) Control group was used by randomly selecting students who came for counseling services on Tuesday and Thursday, and was receiving normal care.

(3) Inclusion criteria by specifying the characteristics according to the specified qualifications.

(3.1) The population studying at Thammasat University, who were experiencing a mental crisis during a period of 2 weeks or more and not more than 6 months and who came for services to receive counseling on mental health problems at the Mental Health Clinic of Nursing Faculty and had a psychological crisis impact score assessed from Adolescent Psychological Crisis Impact Questionnaire [23] level was moderate or higher.

(3.2) All sexes, aged 18-21 years are willing to cooperate in this research.

(4) Exclusion criteria are the subject undergoing treatment or has been diagnosed by a physician with a physical disease or a psychiatric illness.

(5) Criteria for termination of participation in the project discontinuation criteria include less than 80% involvement in the study without being able to do remedial activities.

If found, students who are experiencing a mental crisis within 2 weeks, the researcher will give advice and provide initial assistance, including referrals to receive appropriate care and assistance.

### 2.2 Data Collection Tools

Part 1: general information questionnaire including sex, age, nationality, religion, educational level, current residence, expenses, income, state of indebtedness, family relationships, family support, and relationships with friends

Part 2: The life crisis awareness questionnaire of Intarakamhang and Thongpukdee (2010), consisted of 40 items of which 16 pertained to stress matters, 10 pertained to coping with emotional problems and 14 pertained to adaptation assessment. The questions were formatted as a four-level evaluation scale. This questionnaire passed the tool quality test by finding the confidence value of the whole questionnaire. Specific confidence values were as follows: 16 stress assessment questions had a confidence value of 0.92**;** 10 questions on coping with emotional problems had a confidence value of 0.76**;** and 14 questions on self-adaptation had a confidence value of 0.87 [5, 6]. In addition, researchers experimented with adolescents with characteristics similar to the 10-member sample group. The confidence value was calculated by the Cronbach alpha coefficient correlation formula, arriving at a value of 0.88.

### 2.3 Tools Used in the Experiment

The counseling program for the strengthening of adolescent life crisis management capacity applied the concept of CBT of Beck *et al*. (1979), in conjunction with the concepts of Conner and Butterfield (2003). Altogether, six activities were involved, namely: 1) relationship building, self-analysis, evaluation, and finding problems together with goal setting, and coming to the agreement prior to activity participation**;** 2) analysis of oneself and of others, adolescent life crisis awareness, impacts, crisis-management methods, monitoring homework by use of the internet, and evaluation and finding problems combined with goal setting**;** 3) changing thought behaviors**;** analyzing situations, thoughts, and feelings**;** and techniques of altering thought behaviors**;** 4) analyzing distorted thought patterns, thought stopping and techniques of altering thought behaviors, self-assessment and self-made changes that have occurred**;** 5) self-analysis, self-made changes that have occurred, evaluating, and setting goals in life**;** and 6) group-activity summary, assessing one’s own life crisis awareness, group termination, and finding content-based information [23]. Five qualified individuals then conducted tests to determine the feasibility of these program activities, as well as how appropriate and attractive they were, and the amount of time needed to implement the program before using it with the actual experimental group. They then made the necessary adaptations to ensure its suitability.

### 2.4 Safeguarding the Rights of the Sample Groups

The present research has passed the review of the Human Research Ethics Committee of Thammasat University (Science), Thammasat University (ECScTU), COA number 130/2562, on the 30^th^ day of September of 2019, research project code 056**/**2562. The rights ceded to the sample group have been described in detail by the researchers. In the pursuit of their research, their intentions are to maintain the prerogatives of the sample group members either to accept, refuse, or terminate involvement in the program of research as the group members may so desire. They may consider their decision either to participate in or withdraw from this program of research without affecting their studies, activities, or grades of any sort whatsoever.

## 3 RESULTS

### 3.1 Descriptive Statistical Analysis of the Sample

From an overall analysis of the research results, it was found that the majority of experimental group and control group members were 20 to 22 years of age and that the members of the experimental group fell entirely within 20-22-year age group (M=21.6, SD=0.56). Members of the control group were within 20-23-year age group (M=21.5, SD=0.71). After applying the independent t-test to compare the age-related data of the experimental group with the corresponding data of the control group, it was found that there was no difference between the two groups. The majority (96.65%) of both the experimental group and control group members were female, while only a minority were male. All members (100% of both groups) were Thai nationals. A clear majority (96.70% of the experimental group and 93.30% of the control group) followed the Buddhist religion. The educational level of the majority in the two groups (90% of the experimental group and 80% of the control group) was at the fourth year of study. The majority (80% of the experimental group and 76.70% of the control group) resided in the dormitories. The majority (86.70% of the experimental group and 76.70% of the control group) had their expenses paid by their parents. The majority (73.30% of the experimental group and 70% of the control group) had sufficient income to meet their expenses. Most of their families (60% of the experimental group and 66.70% of the control group) were debt-free. The majority (99.30% of the experimental group and 76.70% of the control group) enjoyed close, intimate relationships with their family members. The majority of members (80% from the experimental group and 73.30% from the control group) were receiving a flexible form of support from their families. Most members (86.70% of the experimental group and 73.30% of the control group) found acceptance in their relationships with friends. From a chi-square personal data analysis, it was found that there were no statistical differences in the results being caused by all factors, as shown in Table **1**.

### 3.2 Inferential Statistical Analysis

1) From an independent t-test comparing the average scores of life crisis awareness of the experimental group with the corresponding average scores of the control group, it was found that there was no difference in life crisis awareness prior to testing between the experimental group and the control group, as shown in Table **2**.

2) A comparative analysis of life crisis awareness scores of the experimental group was performed both before and after testing by use of a dependent t-test. Following entry into the counseling program for the reinforcement of life crisis management, analysis results showed that the life crisis awareness of the experimental group – both before and after testing – was higher (M=132.77, SD=11.03) than it was prior to program entry (M=113.73, SD=14.40), with a statistical significance (t=6.81, p<0.001), as shown in Table **3**.

3) A comparative analysis of life crisis awareness scores between the experimental and control groups was performed both before and after testing by use of an independent t-test. Analysis results showed that the pretest life crisis awareness scores of adolescent students in the experimental group (M=113.73, SD=14.40) and the control group (M=116.53, SD=12.11) did not differ (t=0.81, p=0.41). However, it was found that the life crisis awareness scores of the experimental group that had entered into the counseling program for the strengthening of mental crisis awareness (M=132.77, SD=11.03) were higher than those of the control group whose members had received standard conventional care (M=118.83, SD=10.73), with a statistical significance (t 4.95, p<0.001), as shown in Table **4**.

## 4 DISCUSSION

A counseling program for the strengthening of mental crisis management capacity has been put into effect. A study of its effectiveness on the life crises of adolescent students has shown that the adolescent life crisis awareness in the experimental group following the group’s entry into the counseling program for the strengthening of mental crisis management capacity was at a higher level than it was prior to testing. The higher level was the result of having carried out the six activities of the above-mentioned counseling program, which the researchers developed by applying the theory of CBT of Beck *et al*. (1979), in conjunction with the concepts of Conner and Butterfield (2003) [15, 16]. These activities consisted of these six phases of the program: 1) relationship building and analysis of oneself and of others; 2) mental crisis awareness and understanding, impacts, and crisis management methods; 3) changing thought behaviors and analyzing situations, thoughts, and feelings**;** 4) analyzing distorted thought patterns, thought stopping, self-assessment, and self-made changes that have occurred**;** 5) analyzing the self-made changes that have occurred and setting goals in life; and 6) assessing one’s own life crisis awareness [23]. The result is a life crisis awareness that includes stress awareness, coping with emotional problems, and adaptation assessment for adolescent students who have entered into the counseling program for further strengthening of their mental crisis management capacity [24-26]. These findings are in agreement with the study conducted by Bundasak *et al*. (2019) [27], who discovered that strengthening mental fortitude helps the adolescent to handle stressful situations efficiently and to be prepared to start a new life with a happy outlook. Should the adolescent find himself challenged with yet another stressful situation, the adolescent will learn from it and then draw upon his own human capacity to fix the problem decisively [28-30].

## CONCLUSION

It was discovered, moreover, that adolescent student life crisis awareness in the experimental group, after entry into the counseling program for the strengthening of mental crisis management capacity, was higher than in the control group whose members received standard conventional care. This higher awareness level demonstrates that when the adolescent students in the experimental group participated in the counseling program, they developed a better grasp of their life crises than the adolescent students who did not participate. It was increasingly clear that mental health was largely based on appropriate life experience and conduct and on any corrective actions that must take place early in the development of the individual [31]. This program thus helps the adolescent to confront a mental crisis and to eradicate currents of negative thinking, erroneous thoughts that deviate from reality, and irrational notions that are causing the mental crisis he is experiencing [32, 33]. It could also help them to persevere through a very terrible event that would be psychologically disturbing to the point of his being tormented with a mix of pain, fear, and anxiety. It would upset his learning and carrying on to present-day life to restore a sense of mental balance and live a normal happy life [34-36]. These findings harmonize with the results of educational programs for adolescents that are concerned with the promotion and prevention of mental health problems or mental crises [37, 38]. They include a program for building mental fortitude, a motivational program directed toward achievement, and a vigorous view of the world, an innovative training program including contact sports and counseling, and a cognitive-behavioral program. These programs can help the adolescent efficiently overcome problems that they may be facing that were rooted in twisted thoughts and notions, enabling the adolescent to return to a life of normality, psychological well-being, and happiness once again [39-41].

## RECOMMENDATIONS

1. As regards this counseling program for the strengthening of mental crisis management capacity, it can be used in psychiatric care as a treatment guideline for adolescents in the area of promotion and prevention of mental health problems. It is also applicable to those whose duties consist of caring for adolescents in other groups of educational institutions. They should first start training in the proper use of this program since certain types of activities on their part can affect the mental state of adolescents.
2. A study should be undertaken to monitor life crisis awareness at three and six months following entry into the counseling program for the strengthening of mental crisis management capacity. Such a study would serve the purpose of assessing effective life crisis awareness and endurance.
3. A study should be undertaken of the above-mentioned counseling program so that the results of the study could then be extended for use in other population groups in the future as well.

## Figures and Tables

**Fig. (1):**
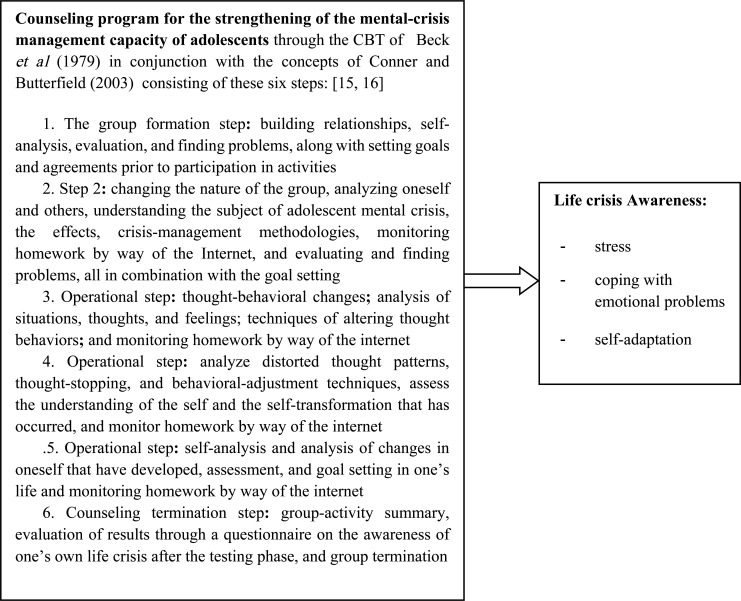
Conceptual framework of the counseling program for the​ strengthening​ of the​ mental-crisis management capacity of adolescence.

**Table 1:** An overall analysis of the control group and the experimental group (n=60).

**General Personal Information**		**Experimental Group ** ** (n= 30) **			** Control Group ** ** (n= 30) **		** Test Value **		** p **	
		** Amount (Percent) **			** Amount (Percent) **					
**Sex:** male		1 (3.35)			1 (3.35)		.15^a^		2.06	
female		29 (96.65)			29 (96.65)					
**Race:** Thai		30 (100)			30 (100)					
**Religion:** Buddhist		29 (96.70)			28 (93.30)		.55		0.35	
Islam		1 (3.30)			2 (6.70)					
**Education level:** 2^nd^ year		0 (0)			2 (6.70)		.31^ a^		2.31	
3^rd^ year		3 (10.00)			4 (13.30)					
4^th^ year		27 (90.00)			24 (80.00)					
**Resided in:** Won house		6 (20.00)			4 (13.30)		.33^ a^		3.42	
Relative‘s house		0 (0)			1 (3.30)					
dormitories		24 (80.00)			23 (76.70)					
other		0 (0)			2 (6.70)					
**Expenses paid:** parents.		26 (86.70)			23 (76.70)		.31^a^		1.00	
scholarship		4 (13.30)			7 (23.30)					
**Income to meet their expenses:** insufficient income		8 (26.70)			9 (30.00)		.77^a^		0.08	
sufficient income		22 (73.30)			21 (70.00)					
**Family debt:** debt-free		18 (60.00)			20 (66.70)		.59^a^		0.28	
In debt		12 (40.00)			10 (33.30)					
**Intimate relationships:** enjoyed close		28 (93.30)			23 (76.70)		.17^a^		3.49	
casual		2 (6.70)			6 (20.00)					
other		0 (0)			1 (3.30)					
**Support from their families:** strict		2 (6.70)			2 (6.70)		.25^a^		5.28	
indulgent		0 (0)			4 (13.30)					
flexible		24 (80.00)			22 (73.30)					
other		1 (3.30)			0 (0)					
indulgent & flexible		3 (10.00)			2 (6.70)					
**Relationships with friends:** acceptance		26 (86.70)			22 (73.30)		.30^a^		3.66	
Some acceptance		2 (6.70)			6 (20.00)					
Minority acceptance		1 (3.30)			2 (6.70)					
other		1 (3.30)			0 (0)					
**age**	**range**		**M(SD)**	**range**		**M(SD)**				
	20-22		21.6 (0.56)	20-23		21.5 (0.71)		.06^b^		0.09

^
a^ = Chi-square test **,** ^b^ = independent t-test

**Table 2:** Comparison of average life crisis awareness scores of the experimental group with the control group prior to testing (n=60).

Average Life Crisis Awareness Scores	Possible Score Range	Experimental Group (n=30)			Control Group (n=30)			df	t	p
		Actual Scores	M	SD	Actual Scores	M	SD			
Prior.to testing	40-160	73-140	113.73	14.40	95-142	116.53	12.11	58	0.81	0.41

**Table 3:** A comparative analysis of the life crisis awareness scores of both before and after testing of the control group and the experimental group (n=60).

Average Life Crisis Awareness Scores	PossiBle Score Range	Prior to Testing			After Testing			df	t	p
		Actual Scores	M	SD	Actual Scores	M	SD			
Control group	40-160	95-142	116.53	12.11	92-137	118.83	10.73	29	0.86	0.39
Experimental group	40-160	73-140	113.73	14.40	105-153	132.77	11.03	29	6.81	0.00*

P value for dependent t-test after comparing pre-and post-treatment scores in each group.

*Statistically significant at p<0.001.

**Table 4:** A comparative analysis of the life crisis awareness scores of the control group with the corresponding scores of the experimental group both before and after testing (n=60).

AveRage Life Crisis Awareness Scores	PossIble Score Range	Experimental Group (n=30)			Control Group (n=30)			df	t	p
		Actual Scores	M	SD	Actual Scores	M	SD			
Prior to testing	40-160	73-140	113.73	14.40	95-142	116.53	12.11	58	0.81	0.41
After testing	40-160	105-153	132.77	11.03	92-137	118.83	10.73	58	4.95	0.00*

P value for independent t-test comparing pre-and post-treatment scores between groups.

*Statistically significant at p<0.001.

## Data Availability

The data that support the findings of this study are available on request from the corresponding author [A.J].
